# 1,2-Bis(dimethyl­amino)-1,2-bis­(2,4,6-triisopropyl­phen­yl)diborane(4)

**DOI:** 10.1107/S1600536810049500

**Published:** 2010-11-30

**Authors:** Holger Braunschweig, Alexander Damme

**Affiliations:** a Institut fuer Anorganische Chemie, Universitaet Wuerzburg, Am Hubland, D-97074 Wuerzburg, Germany

## Abstract

In the mol­ecular structure of the title compound, C_34_H_58_B_2_N_2_, each B atom of the diborane(4) is connected to one dimethyl­amino group and one Tip ligand (Tip = 2,4,6-triisopropyl­phen­yl). These findings indicate that the increased steric demand of the Tip groups exerts influence solely on the B—B separation but not on the overall geometry of the title compound.

## Related literature

For the synthesis of the title compound with 1,2-bis­(di­methyl­amino)-1,2-dichloro­diborane(4) as starting material, see: Hunold (1988 ▶). For 1,2-diaryl-1,2-bis­(dimethyl­amino)­di­boranes(4) (aryl = phenyl or mesit­yl), see: Moezzi *et al.* (1992 ▶) and for dimesityldiboranes(4), see: Hommer *et al.* (1998 ▶).
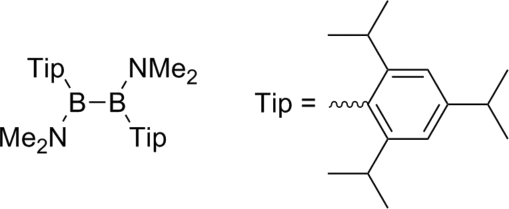


## Experimental

### 

#### Crystal data

C_34_H_58_B_2_N_2_
*M* *_r_* = 516.44
Triclinic, 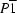

*a* = 9.6066 (19) Å
*b* = 13.919 (3) Å
*c* = 14.015 (3) Å
α = 82.983 (3)°
β = 71.549 (3)°
γ = 78.300 (3)°
*V* = 1737.4 (6) Å^3^
*Z* = 2
Mo *K*α radiation
μ = 0.06 mm^−1^
*T* = 171 K
0.40 × 0.17 × 0.12 mm

#### Data collection

Bruker APEXI CCD diffractometer
Absorption correction: multi-scan (*SADABS*; Bruker, 2008 ▶) *T*_min_ = 0.494, *T*_max_ = 0.745
19559 measured reflections
7202 independent reflections
5554 reflections with *I* > 2σ(*I*)
*R*_int_ = 0.062

#### Refinement

*R*[*F* ^2^ > 2σ(*F* ^2^)] = 0.061
*wR*(*F* ^2^) = 0.173
*S* = 1.03
7202 reflections
359 parameters
H-atom parameters constrained
Δρ_max_ = 0.30 e Å^−3^
Δρ_min_ = −0.24 e Å^−3^

### 

Data collection: *SMART-NT* (Bruker, 1997 ▶); cell refinement: *SAINT-Plus-NT* (Bruker, 1997 ▶); data reduction: *SAINT-Plus-NT*; program(s) used to solve structure: *SHELXS97* (Sheldrick, 2008 ▶); program(s) used to refine structure: *SHELXL97* (Sheldrick, 2008 ▶); molecular graphics: *XP* (Bruker, 1997 ▶); software used to prepare material for publication: *SHELXL97*.

## Supplementary Material

Crystal structure: contains datablocks I, global. DOI: 10.1107/S1600536810049500/br2151sup1.cif

Structure factors: contains datablocks I. DOI: 10.1107/S1600536810049500/br2151Isup2.hkl

Additional supplementary materials:  crystallographic information; 3D view; checkCIF report

## Figures and Tables

**Fig. 1.**
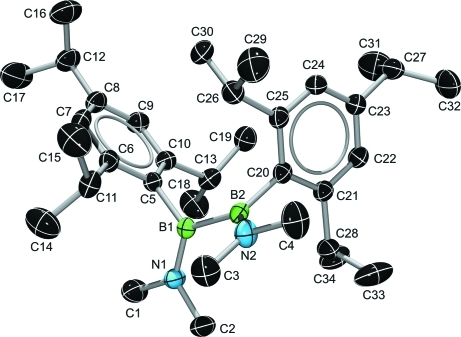
The molecular structure of the title compound showing the atom numbering scheme and displacement ellipsoids for the non-H atoms at the 50% probability level. Hydrogen atoms are omitted for clarity.

## supplementary crystallographic information

### Comment

The B–B bond of 1,2-bis(dimethylamino)-1,2-bis(2,4,6-triisopropylphenyl)diborane(4) is with 1.731 (2) Å slightly longer with respect to those reported previously for phenyl (1.714 (4) Å) and mesityl (1.717 (15) Å) substituted 1,2-bis(dimethylamino)diboranes(4) (Moezzi *et al.*, 1992), but the B–N (about 1.40 Å) and B–C_i_ (about 1.59 Å) bonds are comparable to the corresponding bond lengths of the afore mentioned compounds.

In the case of 1,2-bis(dimethylamino)-1,2-bis(2,4,6-trimethylphenyl)diborane(4) a reaction with MeOH and etheric HCl leads to 1,2-bis(2,4,6-trimethylphenyl)-1,2-di(methoxy)diborane(4), this is not possible with 1,2-bis(dimethylamino)-1,2-bis(2,4,6-triisopropylphenyl)diborane(4) (Hunold, 1988).

### Experimental

A solution of 2,4,6-triisopropylphenyllithium (2.09 g, 7.34 mmol, 2.18 eq) in Et_2_O (25 ml) was added to 1,2-bis(dimethylamino)-1,2-dibromodiborane(4) (0.908 g, 3.36 mmol) dissolved in Et_2_O (15 ml). The reaction mixture was refluxed for 16 h. All volatiles were removed under reduced pressure. The crude product was crystallized from hexane to yield 60% 1,2-bis(dimethylamino)-1,2-bis(2,4,6-triisopropylphenyl)diborane(4) as colourless crystals (1.04 g, 2.01 mmol).

NMR: ^1^H NMR (300.0 K, 500.13 MHz, CDCl_3_): δ = 0.02 (d, ^3^*J*_H–H_ = 6.70 Hz, 6H, C*H_3_*), 0.92 (d, ^3^*J*_H–H_ = 6.85 Hz, 6H, C*H_3_*), 1.12–1.18 (m, 18H, C*H_3_*), 1.22 (d, ^3^*J*_H–H_ = 6.85 Hz, 6H, C*H_3_*), 2.15–2.23 (m, 2H, C*H*), 2.70–2.78 (m, 2H, C*H*), 2.78–2.85 (m, 2H, C*H*), 2.64 (s, 6H, NC*H*_3_), 3.12 (s, 6H, NC*H*_3_), 6.62–6.65 (m, 2H, C*H*_arom._), 6.81–6.83 p.p.m. (m, 2H, C*H*_arom._); ^11^B NMR (296.1 K, 160.46 MHz, CDCl_3_): δ = 49.6 p.p.m.; ^13^C NMR (300.0 K, 160.46 MHz, CDCl_3_): δ = 149.15 (s, *C*_i_), 148.17 (s, *C*_i_), 146.66 (s, *C*_i_), 142.00 (s, *C*_i_), 120.11 (s, *C*H_arom._), 119.41 (s, *C*H_arom._), 43.94 (s, N*C*H_3_), 42.29 (s, N*C*H_3_), 34.36 (s, *C*H), 34.16 (s, *C*H), 33.98 (s, *C*H), 25.35 (s, *C*H_3_), 25.27 (s, *C*H_3_), 24.41 (s, *C*H_3_), 24.13 (s, *C*H_3_), 22.64 p.p.m. (s, *C*H_3_).

Analysis calcd. for C_34_H_58_B_2_N_2_: C, 79.07; H, 11.32; N, 5.42%. Found: C, 78.79; H, 11.21; N, 5.20%.

### Refinement

The H atoms were placed at idealized positions and treated as riding atoms with C–H = 0.98 Å (CH_3_), 1.00 Å (aliphatic CH) and 0.95 Å (aromatic CH). *U*_iso_(H) values were fixed at 1.5 times (for primary H atoms) and 1.2 times (tertiary or aromatic H atoms) *U*_eq_ of the attached C atoms.

### Crystal data

**Table:**

C_34_H_58_B_2_N_2_	*Z* = 2
*M**_r_* = 516.44	*F*(000) = 572
Triclinic, *P*1	*D*_x_ = 0.987 Mg m^−^^3^
Hall symbol: -P 1	Melting point: 192.11 K
*a* = 9.6066 (19) Å	Mo *K*α radiation, λ = 0.71073 Å
*b* = 13.919 (3) Å	Cell parameters from 5619 reflections
*c* = 14.015 (3) Å	θ = 2.2–26.5°
α = 82.983 (3)°	µ = 0.06 mm^−^^1^
β = 71.549 (3)°	*T* = 171 K
γ = 78.300 (3)°	Block, colourless
*V* = 1737.4 (6) Å^3^	0.40 × 0.17 × 0.12 mm

### Data collection

**Table:**

Bruker APEXI CCD diffractometer	7202 independent reflections
Radiation source: sealed tube	5554 reflections with *I* > 2σ(*I*)
graphite	*R*_int_ = 0.062
φ and ω scans	θ_max_ = 26.6°, θ_min_ = 1.5°
Absorption correction: multi-scan (*SADABS*; Bruker, 2008)	*h* = −12→12
*T*_min_ = 0.494, *T*_max_ = 0.745	*k* = −17→17
19559 measured reflections	*l* = −17→17

### Refinement

**Table:**

Refinement on *F*^2^	Primary atom site location: structure-invariant direct methods
Least-squares matrix: full	Secondary atom site location: difference Fourier map
*R*[*F*^2^ > 2σ(*F*^2^)] = 0.061	Hydrogen site location: inferred from neighbouring sites
*wR*(*F*^2^) = 0.173	H-atom parameters constrained
*S* = 1.02	*w* = 1/[σ^2^(*F*_o_^2^) + (0.0841*P*)^2^ + 0.3205*P*] where *P* = (*F*_o_^2^ + 2*F*_c_^2^)/3
7202 reflections	(Δ/σ)_max_ < 0.001
359 parameters	Δρ_max_ = 0.30 e Å^−^^3^
0 restraints	Δρ_min_ = −0.24 e Å^−^^3^

### Special details

**Table:**

Geometry. All e.s.d.'s (except the e.s.d. in the dihedral angle between two l.s. planes) are estimated using the full covariance matrix. The cell e.s.d.'s are taken into account individually in the estimation of e.s.d.'s in distances, angles and torsion angles; correlations between e.s.d.'s in cell parameters are only used when they are defined by crystal symmetry. An approximate (isotropic) treatment of cell e.s.d.'s is used for estimating e.s.d.'s involving l.s. planes.
Refinement. Refinement of *F*^2^ against ALL reflections. The weighted *R*-factor *wR* and goodness of fit *S* are based on *F*^2^, conventional *R*-factors *R* are based on *F*, with *F* set to zero for negative *F*^2^. The threshold expression of *F*^2^ > σ(*F*^2^) is used only for calculating *R*-factors(gt) *etc*. and is not relevant to the choice of reflections for refinement. *R*-factors based on *F*^2^ are statistically about twice as large as those based on *F*, and *R*- factors based on ALL data will be even larger.

### Fractional atomic coordinates and isotropic or equivalent isotropic displacement parameters (Å^2^)

**Table:**

	*x*	*y*	*z*	*U*_iso_*/*U*_eq_	
B1	0.51577 (18)	0.82586 (12)	0.21886 (12)	0.0269 (3)	
B2	0.54148 (19)	0.73592 (12)	0.31158 (12)	0.0280 (3)	
N1	0.57731 (15)	0.91179 (9)	0.20047 (9)	0.0333 (3)	
C1	0.5537 (2)	0.99154 (13)	0.12572 (14)	0.0497 (5)	
H1A	0.4878	0.9756	0.0912	0.057 (6)*	
H1B	0.6496	0.9996	0.0764	0.061 (6)*	
H1C	0.5078	1.0528	0.1596	0.066 (6)*	
C2	0.6729 (2)	0.93496 (13)	0.25401 (13)	0.0430 (4)	
H2A	0.6851	0.8820	0.3052	0.043 (5)*	
H2B	0.6272	0.9969	0.2867	0.057 (6)*	
H2C	0.7705	0.9416	0.2062	0.057 (6)*	
N2	0.68120 (15)	0.68380 (10)	0.31429 (10)	0.0366 (3)	
C3	0.82264 (19)	0.69513 (15)	0.23912 (14)	0.0482 (4)	
H3A	0.8059	0.7490	0.1898	0.042 (5)*	
H3B	0.8663	0.6340	0.2047	0.058 (6)*	
H3C	0.8909	0.7100	0.2723	0.067 (6)*	
C4	0.7037 (2)	0.60721 (15)	0.39242 (15)	0.0552 (5)	
H4A	0.6078	0.6021	0.4433	0.054 (6)*	
H4B	0.7718	0.6243	0.4244	0.088 (8)*	
H4C	0.7466	0.5441	0.3617	0.070 (7)*	
C5	0.41071 (16)	0.81281 (10)	0.15445 (10)	0.0274 (3)	
C6	0.46957 (17)	0.75872 (11)	0.06756 (11)	0.0318 (3)	
C7	0.37787 (19)	0.74638 (12)	0.01237 (12)	0.0375 (4)	
H7	0.4199	0.7105	−0.0466	0.045*	
C8	0.22699 (19)	0.78480 (12)	0.04069 (12)	0.0375 (4)	
C9	0.16922 (18)	0.83731 (12)	0.12656 (12)	0.0365 (4)	
H9	0.0660	0.8645	0.1471	0.044*	
C10	0.25752 (17)	0.85167 (10)	0.18397 (11)	0.0308 (3)	
C11	0.63496 (18)	0.71524 (12)	0.03242 (12)	0.0389 (4)	
H11	0.6743	0.7153	0.0905	0.047*	
C12	0.1276 (2)	0.76690 (14)	−0.01881 (14)	0.0464 (4)	
H12	0.0228	0.7963	0.0175	0.056*	
C13	0.18586 (18)	0.90841 (12)	0.27877 (12)	0.0375 (4)	
H13	0.2637	0.9040	0.3133	0.045*	
C14	0.7191 (2)	0.77944 (16)	−0.05258 (18)	0.0640 (6)	
H14A	0.6975	0.8471	−0.0321	0.096*	
H14B	0.6878	0.7782	−0.1125	0.096*	
H14C	0.8263	0.7545	−0.0681	0.096*	
C15	0.6675 (2)	0.60935 (14)	0.00323 (18)	0.0636 (6)	
H15A	0.6134	0.5688	0.0595	0.095*	
H15B	0.7748	0.5844	−0.0125	0.095*	
H15C	0.6354	0.6067	−0.0560	0.095*	
C16	0.1325 (3)	0.65785 (16)	−0.02416 (18)	0.0636 (6)	
H16A	0.0668	0.6491	−0.0624	0.095*	
H16B	0.0990	0.6275	0.0442	0.095*	
H16C	0.2348	0.6266	−0.0578	0.095*	
C17	0.1674 (3)	0.8170 (2)	−0.12350 (19)	0.0882 (9)	
H17A	0.1013	0.8044	−0.1596	0.132*	
H17B	0.2710	0.7912	−0.1602	0.132*	
H17C	0.1557	0.8880	−0.1185	0.132*	
C18	0.1340 (3)	1.01727 (14)	0.25493 (18)	0.0669 (6)	
H18A	0.0888	1.0508	0.3179	0.100*	
H18B	0.0603	1.0242	0.2185	0.100*	
H18C	0.2196	1.0467	0.2131	0.100*	
C19	0.05621 (19)	0.86431 (14)	0.35297 (13)	0.0449 (4)	
H19A	0.0904	0.7946	0.3685	0.067*	
H19B	−0.0246	0.8708	0.3228	0.067*	
H19C	0.0199	0.8993	0.4151	0.067*	
C20	0.39744 (16)	0.70813 (10)	0.39634 (10)	0.0284 (3)	
C21	0.33107 (17)	0.76128 (11)	0.48375 (11)	0.0309 (3)	
C22	0.20580 (18)	0.73635 (11)	0.55681 (11)	0.0350 (4)	
H22	0.1638	0.7726	0.6155	0.042*	
C23	0.13993 (18)	0.65943 (11)	0.54635 (11)	0.0348 (4)	
C24	0.20400 (19)	0.60811 (11)	0.45993 (11)	0.0356 (4)	
H24	0.1604	0.5555	0.4514	0.043*	
C25	0.33014 (18)	0.63093 (11)	0.38507 (11)	0.0319 (3)	
C26	0.3937 (2)	0.57152 (12)	0.29141 (12)	0.0417 (4)	
H26	0.4759	0.6032	0.2437	0.050*	
C27	−0.0016 (2)	0.63573 (14)	0.62444 (13)	0.0469 (4)	
H27	−0.0167	0.5707	0.6097	0.056*	
C28	0.39924 (19)	0.84595 (12)	0.49932 (12)	0.0382 (4)	
H28	0.4714	0.8629	0.4333	0.046*	
C29	0.4616 (3)	0.46551 (14)	0.31668 (17)	0.0662 (6)	
H29A	0.5352	0.4662	0.3515	0.099*	
H29B	0.5103	0.4315	0.2543	0.099*	
H29C	0.3827	0.4312	0.3604	0.099*	
C30	0.2782 (3)	0.57269 (15)	0.23764 (13)	0.0536 (5)	
H30A	0.2367	0.6409	0.2217	0.080*	
H30B	0.1981	0.5394	0.2814	0.080*	
H30C	0.3255	0.5386	0.1752	0.080*	
C31	−0.1356 (2)	0.7113 (2)	0.6151 (2)	0.0797 (7)	
H31A	−0.2258	0.6945	0.6656	0.120*	
H31B	−0.1453	0.7116	0.5475	0.120*	
H31C	−0.1221	0.7765	0.6261	0.120*	
C32	0.0069 (3)	0.62659 (18)	0.73136 (14)	0.0639 (6)	
H32A	0.0942	0.5780	0.7361	0.096*	
H32B	−0.0835	0.6055	0.7775	0.096*	
H32C	0.0154	0.6904	0.7497	0.096*	
C33	0.4866 (3)	0.81472 (16)	0.5746 (2)	0.0688 (6)	
H33A	0.5601	0.7554	0.5531	0.103*	
H33B	0.4181	0.8009	0.6412	0.103*	
H33C	0.5379	0.8677	0.5781	0.103*	
C34	0.2852 (2)	0.93833 (13)	0.53104 (17)	0.0562 (5)	
H34A	0.2298	0.9578	0.4818	0.084*	
H34B	0.3364	0.9915	0.5342	0.084*	
H34C	0.2158	0.9253	0.5975	0.084*	

### Atomic displacement parameters (Å^2^)

**Table:**

	*U*^11^	*U*^22^	*U*^33^	*U*^12^	*U*^13^	*U*^23^
B1	0.0259 (8)	0.0282 (8)	0.0253 (8)	−0.0020 (6)	−0.0074 (6)	−0.0024 (6)
B2	0.0348 (9)	0.0261 (8)	0.0258 (8)	−0.0041 (6)	−0.0128 (7)	−0.0033 (6)
N1	0.0410 (7)	0.0300 (6)	0.0346 (7)	−0.0105 (5)	−0.0184 (6)	0.0034 (5)
C1	0.0680 (13)	0.0368 (9)	0.0545 (11)	−0.0206 (9)	−0.0322 (10)	0.0147 (8)
C2	0.0491 (10)	0.0434 (10)	0.0461 (10)	−0.0187 (8)	−0.0225 (8)	0.0009 (8)
N2	0.0356 (7)	0.0375 (7)	0.0340 (7)	0.0011 (6)	−0.0132 (6)	0.0026 (5)
C3	0.0330 (9)	0.0562 (11)	0.0502 (11)	0.0012 (8)	−0.0116 (8)	−0.0012 (9)
C4	0.0545 (12)	0.0535 (12)	0.0497 (11)	0.0102 (9)	−0.0223 (9)	0.0120 (9)
C5	0.0316 (8)	0.0258 (7)	0.0262 (7)	−0.0060 (6)	−0.0115 (6)	0.0027 (5)
C6	0.0369 (8)	0.0313 (8)	0.0276 (7)	−0.0051 (6)	−0.0121 (6)	0.0013 (6)
C7	0.0458 (9)	0.0415 (9)	0.0286 (8)	−0.0067 (7)	−0.0153 (7)	−0.0053 (6)
C8	0.0429 (9)	0.0414 (9)	0.0347 (8)	−0.0104 (7)	−0.0197 (7)	0.0007 (7)
C9	0.0329 (8)	0.0380 (9)	0.0416 (9)	−0.0043 (7)	−0.0169 (7)	−0.0011 (7)
C10	0.0331 (8)	0.0277 (7)	0.0333 (8)	−0.0052 (6)	−0.0132 (6)	−0.0001 (6)
C11	0.0393 (9)	0.0453 (9)	0.0318 (8)	0.0012 (7)	−0.0135 (7)	−0.0081 (7)
C12	0.0492 (10)	0.0537 (11)	0.0471 (10)	−0.0122 (8)	−0.0278 (8)	−0.0025 (8)
C13	0.0336 (8)	0.0389 (9)	0.0417 (9)	−0.0008 (7)	−0.0142 (7)	−0.0107 (7)
C14	0.0422 (11)	0.0548 (12)	0.0795 (15)	−0.0053 (9)	0.0015 (10)	−0.0042 (11)
C15	0.0574 (13)	0.0438 (11)	0.0723 (14)	0.0034 (9)	−0.0005 (11)	−0.0108 (10)
C16	0.0714 (14)	0.0623 (13)	0.0758 (15)	−0.0225 (11)	−0.0409 (12)	−0.0053 (11)
C17	0.119 (2)	0.108 (2)	0.0763 (16)	−0.0654 (18)	−0.0728 (16)	0.0400 (15)
C18	0.0763 (15)	0.0375 (10)	0.0713 (14)	−0.0027 (10)	−0.0018 (12)	−0.0118 (10)
C19	0.0394 (9)	0.0502 (10)	0.0413 (9)	−0.0012 (8)	−0.0090 (7)	−0.0076 (8)
C20	0.0355 (8)	0.0241 (7)	0.0269 (7)	−0.0026 (6)	−0.0137 (6)	0.0009 (5)
C21	0.0369 (8)	0.0275 (7)	0.0298 (8)	−0.0050 (6)	−0.0120 (6)	−0.0026 (6)
C22	0.0412 (9)	0.0355 (8)	0.0288 (8)	−0.0079 (7)	−0.0081 (7)	−0.0083 (6)
C23	0.0392 (9)	0.0358 (8)	0.0306 (8)	−0.0107 (7)	−0.0093 (7)	−0.0022 (6)
C24	0.0473 (9)	0.0303 (8)	0.0331 (8)	−0.0142 (7)	−0.0134 (7)	−0.0011 (6)
C25	0.0427 (9)	0.0260 (7)	0.0277 (7)	−0.0056 (6)	−0.0117 (6)	−0.0020 (6)
C26	0.0572 (11)	0.0342 (8)	0.0316 (8)	−0.0152 (8)	−0.0041 (7)	−0.0069 (7)
C27	0.0484 (10)	0.0499 (10)	0.0416 (10)	−0.0225 (8)	−0.0006 (8)	−0.0117 (8)
C28	0.0440 (9)	0.0371 (8)	0.0348 (8)	−0.0148 (7)	−0.0067 (7)	−0.0078 (7)
C29	0.0898 (17)	0.0394 (10)	0.0592 (13)	0.0027 (10)	−0.0113 (12)	−0.0176 (9)
C30	0.0842 (15)	0.0497 (11)	0.0352 (9)	−0.0282 (10)	−0.0178 (9)	−0.0072 (8)
C31	0.0397 (12)	0.1007 (19)	0.0950 (18)	−0.0201 (12)	−0.0103 (12)	−0.0055 (15)
C32	0.0706 (14)	0.0744 (14)	0.0379 (10)	−0.0280 (11)	0.0071 (9)	−0.0076 (9)
C33	0.0684 (14)	0.0534 (12)	0.1082 (19)	−0.0114 (10)	−0.0560 (14)	−0.0135 (12)
C34	0.0718 (14)	0.0369 (9)	0.0727 (13)	−0.0073 (9)	−0.0361 (11)	−0.0159 (9)

### Geometric parameters (Å, °)

**Table:**

B1—N1	1.401 (2)	C16—H16C	0.9800
B1—C5	1.599 (2)	C17—H17A	0.9800
B1—B2	1.731 (2)	C17—H17B	0.9800
B2—N2	1.400 (2)	C17—H17C	0.9800
B2—C20	1.594 (2)	C18—H18A	0.9800
N1—C2	1.458 (2)	C18—H18B	0.9800
N1—C1	1.465 (2)	C18—H18C	0.9800
C1—H1A	0.9800	C19—H19A	0.9800
C1—H1B	0.9800	C19—H19B	0.9800
C1—H1C	0.9800	C19—H19C	0.9800
C2—H2A	0.9800	C20—C25	1.409 (2)
C2—H2B	0.9800	C20—C21	1.411 (2)
C2—H2C	0.9800	C21—C22	1.386 (2)
N2—C3	1.456 (2)	C21—C28	1.527 (2)
N2—C4	1.468 (2)	C22—C23	1.393 (2)
C3—H3A	0.9800	C22—H22	0.9500
C3—H3B	0.9800	C23—C24	1.385 (2)
C3—H3C	0.9800	C23—C27	1.519 (2)
C4—H4A	0.9800	C24—C25	1.393 (2)
C4—H4B	0.9800	C24—H24	0.9500
C4—H4C	0.9800	C25—C26	1.527 (2)
C5—C10	1.406 (2)	C26—C30	1.523 (3)
C5—C6	1.409 (2)	C26—C29	1.535 (3)
C6—C7	1.389 (2)	C26—H26	1.0000
C6—C11	1.523 (2)	C27—C32	1.515 (3)
C7—C8	1.385 (2)	C27—C31	1.518 (3)
C7—H7	0.9500	C27—H27	1.0000
C8—C9	1.385 (2)	C28—C33	1.519 (3)
C8—C12	1.523 (2)	C28—C34	1.520 (3)
C9—C10	1.395 (2)	C28—H28	1.0000
C9—H9	0.9500	C29—H29A	0.9800
C10—C13	1.523 (2)	C29—H29B	0.9800
C11—C14	1.515 (3)	C29—H29C	0.9800
C11—C15	1.522 (3)	C30—H30A	0.9800
C11—H11	1.0000	C30—H30B	0.9800
C12—C17	1.513 (3)	C30—H30C	0.9800
C12—C16	1.519 (3)	C31—H31A	0.9800
C12—H12	1.0000	C31—H31B	0.9800
C13—C18	1.527 (3)	C31—H31C	0.9800
C13—C19	1.531 (2)	C32—H32A	0.9800
C13—H13	1.0000	C32—H32B	0.9800
C14—H14A	0.9800	C32—H32C	0.9800
C14—H14B	0.9800	C33—H33A	0.9800
C14—H14C	0.9800	C33—H33B	0.9800
C15—H15A	0.9800	C33—H33C	0.9800
C15—H15B	0.9800	C34—H34A	0.9800
C15—H15C	0.9800	C34—H34B	0.9800
C16—H16A	0.9800	C34—H34C	0.9800
C16—H16B	0.9800		
			
N1—B1—C5	118.26 (12)	C12—C17—H17A	109.5
N1—B1—B2	123.11 (13)	C12—C17—H17B	109.5
C5—B1—B2	118.57 (12)	H17A—C17—H17B	109.5
N2—B2—C20	118.14 (13)	C12—C17—H17C	109.5
N2—B2—B1	123.73 (13)	H17A—C17—H17C	109.5
C20—B2—B1	118.08 (12)	H17B—C17—H17C	109.5
B1—N1—C2	124.67 (13)	C13—C18—H18A	109.5
B1—N1—C1	124.99 (13)	C13—C18—H18B	109.5
C2—N1—C1	110.35 (13)	H18A—C18—H18B	109.5
N1—C1—H1A	109.5	C13—C18—H18C	109.5
N1—C1—H1B	109.5	H18A—C18—H18C	109.5
H1A—C1—H1B	109.5	H18B—C18—H18C	109.5
N1—C1—H1C	109.5	C13—C19—H19A	109.5
H1A—C1—H1C	109.5	C13—C19—H19B	109.5
H1B—C1—H1C	109.5	H19A—C19—H19B	109.5
N1—C2—H2A	109.5	C13—C19—H19C	109.5
N1—C2—H2B	109.5	H19A—C19—H19C	109.5
H2A—C2—H2B	109.5	H19B—C19—H19C	109.5
N1—C2—H2C	109.5	C25—C20—C21	117.84 (13)
H2A—C2—H2C	109.5	C25—C20—B2	120.87 (12)
H2B—C2—H2C	109.5	C21—C20—B2	121.27 (13)
B2—N2—C3	125.20 (13)	C22—C21—C20	120.65 (13)
B2—N2—C4	124.01 (14)	C22—C21—C28	119.45 (13)
C3—N2—C4	110.75 (14)	C20—C21—C28	119.89 (13)
N2—C3—H3A	109.5	C21—C22—C23	121.62 (14)
N2—C3—H3B	109.5	C21—C22—H22	119.2
H3A—C3—H3B	109.5	C23—C22—H22	119.2
N2—C3—H3C	109.5	C24—C23—C22	117.69 (14)
H3A—C3—H3C	109.5	C24—C23—C27	121.01 (14)
H3B—C3—H3C	109.5	C22—C23—C27	121.24 (14)
N2—C4—H4A	109.5	C23—C24—C25	122.26 (14)
N2—C4—H4B	109.5	C23—C24—H24	118.9
H4A—C4—H4B	109.5	C25—C24—H24	118.9
N2—C4—H4C	109.5	C24—C25—C20	119.92 (13)
H4A—C4—H4C	109.5	C24—C25—C26	119.26 (13)
H4B—C4—H4C	109.5	C20—C25—C26	120.82 (13)
C10—C5—C6	118.13 (13)	C30—C26—C25	112.10 (15)
C10—C5—B1	121.56 (13)	C30—C26—C29	110.65 (16)
C6—C5—B1	120.28 (13)	C25—C26—C29	111.51 (14)
C7—C6—C5	120.25 (14)	C30—C26—H26	107.4
C7—C6—C11	119.63 (14)	C25—C26—H26	107.4
C5—C6—C11	120.11 (13)	C29—C26—H26	107.4
C8—C7—C6	121.97 (15)	C32—C27—C31	110.46 (18)
C8—C7—H7	119.0	C32—C27—C23	113.23 (16)
C6—C7—H7	119.0	C31—C27—C23	110.64 (16)
C7—C8—C9	117.65 (14)	C32—C27—H27	107.4
C7—C8—C12	120.96 (15)	C31—C27—H27	107.4
C9—C8—C12	121.36 (15)	C23—C27—H27	107.4
C8—C9—C10	122.21 (15)	C33—C28—C34	110.25 (15)
C8—C9—H9	118.9	C33—C28—C21	110.89 (14)
C10—C9—H9	118.9	C34—C28—C21	113.31 (14)
C9—C10—C5	119.79 (14)	C33—C28—H28	107.4
C9—C10—C13	119.37 (14)	C34—C28—H28	107.4
C5—C10—C13	120.84 (13)	C21—C28—H28	107.4
C14—C11—C15	110.69 (15)	C26—C29—H29A	109.5
C14—C11—C6	111.02 (14)	C26—C29—H29B	109.5
C15—C11—C6	113.97 (15)	H29A—C29—H29B	109.5
C14—C11—H11	106.9	C26—C29—H29C	109.5
C15—C11—H11	106.9	H29A—C29—H29C	109.5
C6—C11—H11	106.9	H29B—C29—H29C	109.5
C17—C12—C16	110.57 (19)	C26—C30—H30A	109.5
C17—C12—C8	111.54 (16)	C26—C30—H30B	109.5
C16—C12—C8	111.67 (15)	H30A—C30—H30B	109.5
C17—C12—H12	107.6	C26—C30—H30C	109.5
C16—C12—H12	107.6	H30A—C30—H30C	109.5
C8—C12—H12	107.6	H30B—C30—H30C	109.5
C10—C13—C18	112.27 (15)	C27—C31—H31A	109.5
C10—C13—C19	112.46 (13)	C27—C31—H31B	109.5
C18—C13—C19	109.82 (15)	H31A—C31—H31B	109.5
C10—C13—H13	107.3	C27—C31—H31C	109.5
C18—C13—H13	107.3	H31A—C31—H31C	109.5
C19—C13—H13	107.3	H31B—C31—H31C	109.5
C11—C14—H14A	109.5	C27—C32—H32A	109.5
C11—C14—H14B	109.5	C27—C32—H32B	109.5
H14A—C14—H14B	109.5	H32A—C32—H32B	109.5
C11—C14—H14C	109.5	C27—C32—H32C	109.5
H14A—C14—H14C	109.5	H32A—C32—H32C	109.5
H14B—C14—H14C	109.5	H32B—C32—H32C	109.5
C11—C15—H15A	109.5	C28—C33—H33A	109.5
C11—C15—H15B	109.5	C28—C33—H33B	109.5
H15A—C15—H15B	109.5	H33A—C33—H33B	109.5
C11—C15—H15C	109.5	C28—C33—H33C	109.5
H15A—C15—H15C	109.5	H33A—C33—H33C	109.5
H15B—C15—H15C	109.5	H33B—C33—H33C	109.5
C12—C16—H16A	109.5	C28—C34—H34A	109.5
C12—C16—H16B	109.5	C28—C34—H34B	109.5
H16A—C16—H16B	109.5	H34A—C34—H34B	109.5
C12—C16—H16C	109.5	C28—C34—H34C	109.5
H16A—C16—H16C	109.5	H34A—C34—H34C	109.5
H16B—C16—H16C	109.5	H34B—C34—H34C	109.5
			
N1—B1—B2—N2	−60.4 (2)	C7—C8—C12—C16	57.7 (2)
C5—B1—B2—N2	122.56 (16)	C9—C8—C12—C16	−120.30 (19)
N1—B1—B2—C20	121.99 (16)	C9—C10—C13—C18	−68.8 (2)
C5—B1—B2—C20	−55.08 (17)	C5—C10—C13—C18	111.88 (18)
C5—B1—N1—C2	178.94 (14)	C9—C10—C13—C19	55.62 (19)
B2—B1—N1—C2	1.9 (2)	C5—C10—C13—C19	−123.66 (15)
C5—B1—N1—C1	−0.3 (2)	N2—B2—C20—C25	−85.40 (18)
B2—B1—N1—C1	−177.42 (15)	B1—B2—C20—C25	92.38 (16)
C20—B2—N2—C3	175.60 (15)	N2—B2—C20—C21	96.14 (17)
B1—B2—N2—C3	−2.0 (2)	B1—B2—C20—C21	−86.08 (17)
C20—B2—N2—C4	−2.1 (2)	C25—C20—C21—C22	1.4 (2)
B1—B2—N2—C4	−179.74 (15)	B2—C20—C21—C22	179.95 (13)
N1—B1—C5—C10	−85.85 (18)	C25—C20—C21—C28	−179.70 (14)
B2—B1—C5—C10	91.36 (16)	B2—C20—C21—C28	−1.2 (2)
N1—B1—C5—C6	96.22 (17)	C20—C21—C22—C23	−0.8 (2)
B2—B1—C5—C6	−86.57 (17)	C28—C21—C22—C23	−179.69 (15)
C10—C5—C6—C7	1.2 (2)	C21—C22—C23—C24	0.0 (2)
B1—C5—C6—C7	179.19 (14)	C21—C22—C23—C27	−177.07 (15)
C10—C5—C6—C11	179.92 (13)	C22—C23—C24—C25	0.2 (2)
B1—C5—C6—C11	−2.1 (2)	C27—C23—C24—C25	177.26 (15)
C5—C6—C7—C8	−0.9 (2)	C23—C24—C25—C20	0.5 (2)
C11—C6—C7—C8	−179.67 (15)	C23—C24—C25—C26	−179.50 (15)
C6—C7—C8—C9	0.4 (2)	C21—C20—C25—C24	−1.3 (2)
C6—C7—C8—C12	−177.61 (15)	B2—C20—C25—C24	−179.78 (13)
C7—C8—C9—C10	−0.2 (2)	C21—C20—C25—C26	178.71 (14)
C12—C8—C9—C10	177.80 (15)	B2—C20—C25—C26	0.2 (2)
C8—C9—C10—C5	0.5 (2)	C24—C25—C26—C30	56.03 (19)
C8—C9—C10—C13	−178.74 (14)	C20—C25—C26—C30	−123.95 (16)
C6—C5—C10—C9	−1.0 (2)	C24—C25—C26—C29	−68.7 (2)
B1—C5—C10—C9	−178.97 (13)	C20—C25—C26—C29	111.36 (18)
C6—C5—C10—C13	178.28 (13)	C24—C23—C27—C32	133.00 (18)
B1—C5—C10—C13	0.3 (2)	C22—C23—C27—C32	−50.0 (2)
C7—C6—C11—C14	79.3 (2)	C24—C23—C27—C31	−102.4 (2)
C5—C6—C11—C14	−99.42 (18)	C22—C23—C27—C31	74.6 (2)
C7—C6—C11—C15	−46.5 (2)	C22—C21—C28—C33	76.2 (2)
C5—C6—C11—C15	134.77 (16)	C20—C21—C28—C33	−102.66 (18)
C7—C8—C12—C17	−66.6 (2)	C22—C21—C28—C34	−48.4 (2)
C9—C8—C12—C17	115.4 (2)	C20—C21—C28—C34	132.74 (16)
